# INTERACTIONS BETWEEN FRAILTY AND SOCIAL VULNERABILITY ON THE SURVIVAL AND HEALTH TRANSITIONS OF OLDER ADULTS

**DOI:** 10.1093/geroni/igae098.2765

**Published:** 2024-12-31

**Authors:** Heng Wu, Xiaowei Song, Kenneth Rockwood

**Affiliations:** Dalhousie University, Halifax, Nova Scotia, Canada; Fraser Health Authority, Surrey, British Columbia, Canada; Dalhousie University, Halifax, Nova Scotia, Canada

## Abstract

Frailty, an age-related state, is dominated by declining health over time, although stabilization and improvement can occur. We investigated how the accumulation of physical and social deficits interacts in health transitions and survival. Data from participants aged 65+ years in the Chinese Longitudinal Health Longevity Survey (CLHLS) 2005 cohort were analyzed (n=15613; women=57%; mean age=86.2±11.7). Participants were followed in 2008, 2011, 2014, and 2018, with their health and survival status reassessed each time. We constructed both a 47-item frailty index (FI) and a 26-item social vulnerability index (SVI). The FI included domains of morbidities, cognition, lifestyle, and disabilities; the SVI included World Health Organization-defined social determinants of health as – i.e., education, social security and inclusion, work/life/dwelling/services conditions, and early childhood development. At each follow-up, the FI strongly affected survival in a Logistic Regression model with baseline age, FI, SVI, and sex as covariates (e.g., a 1% FI increase for 14-year mortality: Odds Ratio OR=1.07, 95% CI=1.06-1.08). The SVI showed a marginally significant effect on 3-year survival (e.g., a 1% SVI increase, OR=1.01, 95% CI=1.00-1.01), and diminished at longer follow-ups. The FI also affected health transitions (Figure 1A), as did the SVI, albeit to a lesser extent (Figure 1B). In health transitions and survival of older adults, frailty increases risk and often interacts with social vulnerability. Our study underscores why we should characterize both physical and social factors to understand health and its dynamics in aging.

